# Gumbel based p-value approximations for spatial scan statistics

**DOI:** 10.1186/1476-072X-9-61

**Published:** 2010-12-17

**Authors:** Allyson M Abrams, Ken Kleinman, Martin Kulldorff

**Affiliations:** 1 Department of Population Medicine, Harvard Medical School and Harvard Pilgrim Health Care Institute, Boston, USA

## Abstract

**Background:**

The spatial and space-time scan statistics are commonly applied for the detection of geographical disease clusters. Monte Carlo hypothesis testing is typically used to test whether the geographical clusters are statistically significant as there is no known way to calculate the null distribution analytically. In Monte Carlo hypothesis testing, simulated random data are generated multiple times under the null hypothesis, and the p-value is *r/(R + 1)*, where *R *is the number of simulated random replicates of the data and *r *is the rank of the test statistic from the real data compared to the same test statistics calculated from each of the random data sets. A drawback to this powerful technique is that each additional digit of p-value precision requires ten times as many replicated datasets, and the additional processing can lead to excessive run times.

**Results:**

We propose a new method for obtaining more precise p-values with a given number of replicates. The collection of test statistics from the random replicates is used to estimate the true distribution of the test statistic under the null hypothesis by fitting a continuous distribution to these observations. The choice of distribution is critical, and for the spatial and space-time scan statistics, the extreme value Gumbel distribution performs very well while the gamma, normal and lognormal distributions perform poorly. From the fitted Gumbel distribution, we show that it is possible to estimate the analytical p-value with great precision even when the test statistic is far out in the tail beyond any of the test statistics observed in the simulated replicates. In addition, Gumbel-based rejection probabilities have smaller variability than Monte Carlo-based rejection probabilities, suggesting that the proposed approach may result in greater power than the true Monte Carlo hypothesis test for a given number of replicates.

**Conclusions:**

For large data sets, it is often advantageous to replace computer intensive Monte Carlo hypothesis testing with this new method of fitting a Gumbel distribution to random data sets generated under the null, in order to reduce computation time and obtain much more precise p-values and slightly higher statistical power.

## Background

### Introduction

Geographic cluster detection and evaluation are important in disease surveillance. One frequently used method for cluster detection is the spatial scan statistic [1-3] and the related space-time scan statistic [4]. This method has been used to study the geography of infectious diseases such as malaria [5], vector borne diseases such as West Nile Virus [6], many different forms of cancer [7-11], low birth weight [12], syndromic surveillance [13-17], and bovine spongiform encephalopathy [18], among many other diseases.

The spatial scan statistic is found by moving a scanning window across the geographical region of interest, generating a large collection of window locations and sizes that meet pre-defined criteria. A likelihood ratio is calculated for the data corresponding to each window location and size and the spatial scan statistic is the maximum of these likelihood ratios. The window location and size with the maximum likelihood ratio is the most likely cluster; that is, the cluster that is least likely to have occurred by chance [1,2]. Except for the simplest scenarios, there is no known closed-form theoretical distribution for the spatial scan statistic. Therefore, p-values for scan statistics are usually obtained using Monte Carlo hypothesis testing [19].

In Monte Carlo hypothesis testing, a large number of random replicates of the observed data are generated under the null hypothesis. Monte Carlo p-values are asymptotically equivalent to p-values from exact permutation tests as the number of random replicates increases, but the key property of Monte Carlo hypothesis testing p-values is that they maintain the correct alpha level, exactly, as long as the number of replicates plus one is a multiple of 1/α [19-21]. Monte Carlo hypothesis testing can therefore be useful when theoretical distributions are unknown and the number of permutations prohibits a full enumeration. One major drawback to the approach is that small p-values can only be obtained through a very large number of Monte Carlo replicates, which may be computer intensive and time consuming. For the spatial and space-time scan statistics, Monte Carlo hypothesis testing requires the calculation of the likelihood ratio for each location and size of the scanning window, for each replicated data set. Thus, the approach can be computer intensive for very large data sets.

In disease surveillance, the space-time scan statistic is sometimes calculated on a daily basis, to continuously monitor a disease in near real-time [13,22]. These clusters may then be reported to local, state, or federal public health officials for potential investigation. Using a conventional 0.05 α-level would on average result in one false rejection of the null hypothesis every 20 days. Because of limited resources, health officials are not able to investigate a lot of false alarms [13,22]. To control the number of false rejections at a more tenable level, one might instead use an α-level of 1/365 = 0.00274 or 1/3650 = 0.000274, corresponding to one expected false positive every year or every ten years, respectively, for daily analyses. So, instead of 999 replicates for an alpha level of 0.05, we may want to use 99,999 replicates or more for an alpha level of 0.000274, keeping approximately the same ratio. If multiple diseases are under surveillance, this may require a high computational burden with millions of random replicates to be simulated each day when Monte Carlo hypothesis testing is used.

In this article, we propose a way to do hypothesis testing for very small alpha levels with fewer calculations. The approach we take is to find a distribution which closely approximates the distribution of the test statistics that were generated under the null hypothesis, which themselves reflect the distribution of the scan statistic under the null. To do this we generate a relatively small number of random simulated replicates under the null hypothesis. We then use them to estimate parameters for a distribution with a well-characterized functional form. If this distribution fits the sample distribution well, we can use it as an estimate of the distribution of the spatial or space-time scan statistic under the null and use it to generate arbitrarily small p-values. Because we are interested in small p-values, it is particularly important that the estimate is good in the tail of the distribution.

We note that although this paper is focused on the spatial and space-time scan statistics, the general methodology that we propose in this article can easily be applied to other test statistics that rely on Monte Carlo hypothesis testing.

### Scan statistics

The spatial scan statistic is used to identify potentially unusual clustering of events on a map. Events may, for example, be cases of disease incidence, prevalence or mortality. Suppose that there are *p *geographical coordinate pairs marked on a map, each representing a region. The analysis is conditioned on the total number of events, and under the null hypothesis, each event is independently and randomly located in a region with probability proportional to the population in the region, or to some covariate-adjusted population based denominator. How best to adjust for covariates is a critical issue which we do not consider in this paper.

We look at all unique subsets of events that lie within a collection of scanning windows to detect clusters. Although any shape scanning window may be used, we use circles throughout this paper. Consider all circles, *C*_*i,r*_, where *i *= 1,..., *p *indicates the coordinates around which a circle may be centered, and *r *indicates its radius, which ranges from 0 to some pre-specified maximum. Based on the observed and expected number of events inside and outside the circle, calculate the likelihood ratio for each distinct circle [1,2]. The circle with the maximum likelihood ratio is the most likely cluster, that is, the cluster that is least likely to have occurred by chance. For computational simplicity, the logarithm of the likelihood ratio is typically used instead of the ratio itself, and the log-likelihood ratio associated with this circle is defined as the *scan statistic*. Likelihoods can be calculated under different probability models, such as binomial or Poisson.

The space-time scan statistic is analogously used to identify clusters in regions of space and time. Envisioning each discrete moment of time as a separate map, and the set of times as a stack of maps, the circles mentioned above can extend through the maps, making cylinders that are the potential clusters. The cylinder with the maximum likelihood ratio is the most likely cluster, and its log-likelihood ratio is the space-time scan statistic. In space-time models, we consider Poisson as well as space-time permutation-based probability models [4,23].

For this study, we used the SaTScan™ [24] statistical software program, which calculates the scan statistic and implements Monte Carlo hypothesis testing to calculate a p-value. SaTScan™ allows the user to vary many parameters including the maximum cluster size, the probability model, and the number of Monte Carlo replicates.

### Monte Carlo hypothesis testing

When the underlying distribution for the test statistic is unknown it is not possible to calculate a standard analytical p-value. When it is still possible, however, to generate data under the null hypothesis, then Monte Carlo hypothesis testing can be used to calculate Monte Carlo based p-values, as proposed by Dwass [19]. To do this, one first calculates the test statistic from the real data. Then, a large number of random data sets are generated according to the null hypothesis, and the test statistic is calculated for each of these data sets. If one creates *R *random replicates of the data and *r*-1 of those replicates have a test statistic which is greater than or equal to the test statistic from the real data, so that *r *is the rank of the test statistics among the real data, then the Monte Carlo based p-value of the observed test statistic is *r/(1+R)*. If the test statistic from the real data set is among the highest 5 percent from the random data sets, then we can reject the null hypothesis at the α = 0.05 level of statistical significance.

As pointed out by several statisticians [19-21,25], a nice feature of Monte Carlo hypothesis testing is that the correct α-level can be maintained exactly. This is simply done by choosing *R *so that *α(1+R) *is an integer. For example, if *α *= 0.05, then the probability to reject the null hypothesis is exactly 0.05 when R = 19, 99, 999, or 9999 random replicates. Following Bernard [19], suppose R = 19. Under the null hypothesis, the one real and 19 random data sets are generated in exactly the same way, so they are all generated from the same probability distribution. This, in turn, means that the ordering of the 20 test statistics is completely random, so that any single one is equally likely to be the highest, 2^nd ^highest, 3^rd ^highest, and so on, as well as equally likely to be the lowest. Hence, under the null hypothesis, the probability that the test statistic from the real data set has the highest value is 1/20 = 0.05, exactly. If it does have the highest test statistic, the Monte Carlo based p-value is p = r/(1 + R) = 1/(1 + 19) = 1/20 = 0.05, and since p ≤ *α *= 0.05, the null hypothesis is rejected.

Since the correct alpha level is maintained exactly whether R is small or large, one may think that the choice does not matter, but that is not the case, as fewer replicates means lower statistical power [25-27]. Hence, more replicates are always better. For *α *= 0.05, 999 replicates gives very good power, but for smaller alpha levels, an increasingly higher number is needed [21,25].

One drawback with Monte Carlo hypothesis testing is that the p-value can never be smaller than 1/(1 + R). For example, with R = 999, the p-value is never less than 0.001. In most applications, with a 0.01 or 0.05 α-level, that is not a problem, as it is not necessary to differentiate between p-values of, say, 0.001 and 0.00001. A relatively small number of replicates will be sufficient. However, in the context of daily analyses in real-time disease surveillance, a cluster with p ≤ 0.05 will by chance happen once every 20 days, on average. That is too often, and the goal is to detect clusters of disease that are very unusual, and only the most unusual clusters will be investigated further. P-values on the order of 0.0001 or even smaller may be required before an investigation is launched. These p-values require at least 9999 Monte Carlo replicates and even more are needed to ensure good statistical power [21,25]. The number of Monte Carlo replicates required is determined by the desired precision of the p-value, and each additional decimal place requires 10 times the number of Monte Carlo replicates and hence about 10 times the computing time.

### The Gumbel distribution

The spatial scan statistic described above is the maximum value taken over many circle locations and sizes, so the collection of these statistics generated from the Monte Carlo replicates is a distribution of maximum values. Since our technique involves finding a distribution which closely matches the distribution of the replicated statistics, it is natural to consider one of the extreme value distributions as one possible candidate to approximate the desired distribution. The Gumbel distribution is a distribution of extreme values, either maxima or minima. Here, we limit ourselves to distributions of maxima since the scan statistic is a maximum. The cdf for the Gumbel distribution of maxima is $G(x)=e^{-e^{-\frac{x-\mu }{\beta }}}$ and the pdf is $g(x)=\frac{1}{\beta }e^{-\frac{x-\mu }{\beta }}e^{-e^{-\frac{x-\mu }{\beta }}}$. Both *μ *and *β *can be estimated from a sample of observations using method of moments estimators as follows:

$$\overset{\wedge }{\beta }=\frac{s\sqrt{6}}{\pi }$$

$$\overset{\wedge }{\mu }=\bar{X}-0.5772\overset{\wedge }{\beta }$$

where $\bar{X}$ is the sample mean and *s *is the sample standard deviation [28,29].

## Methods

To evaluate whether it is possible to obtain approximate small p-values with only a limited number of Monte Carlo replicates, we performed computer simulations fitting different probability distributions to the sample test statistics from the random data sets generated under the null. For our baseline set-up, we use a map of 245 counties and county equivalents in the Northeast United States, with each county represented by its census-defined centroid [24]. Under the null hypothesis, the number of cases in each county is Poisson distributed. Conditioning on a total of 600 cases, the cases were randomly and independently assigned to a county with probability proportional to the 1994 female population in that county [30]. The maximum circle size of the scan statistic was set to 50% of the population.

First, we generated 100,000,000 Monte Carlo replicates of the data under the null hypothesis. The maximum log-likelihood ratio among all distinct circles is the statistic reported from each replicate. These 100,000,000 statistics generated our "gold standard" distribution of log-likelihood ratios, which we treat as if it were the actual distribution of the statistic under the null. Using this distribution, we find the 'true' log-likelihood ratio corresponding to a given α-level by finding the log-likelihood ratio for which the rank divided by 100,000,000 gives the desired α-level. For example, the log-likelihood ratio with a rank of 1,000,000 corresponds to an α-level of 0.01, since 1,000,000/100,000,000 = 0.01.

Using the same parameter settings, we also generated sets of 999 Monte Carlo replicates of the data. We used the 999 maximum log-likelihood ratios obtained from the Monte Carlo replicates to fit normal, gamma, lognormal and Gumbel distributions to the data to see if any of them would approximate the true distribution of these log-likelihood ratios. The first three were chosen because they are three of the most commonly used continuous distributions, without any deeper rationale. The extreme value Gumbel distribution was chosen since the test statistic is a maximum taken over many possible circles.

**Figure 1:**
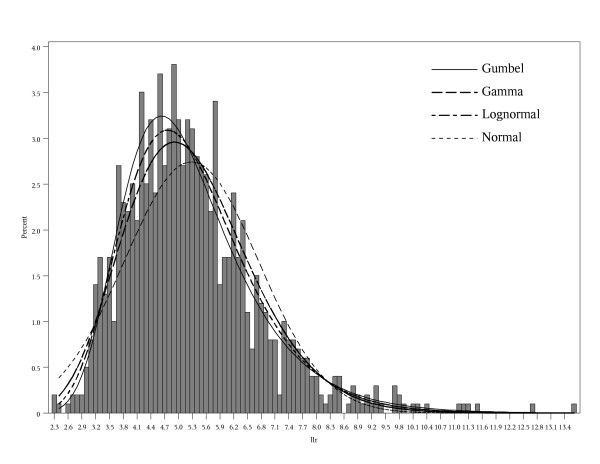
Histogram of 999 log-likelihoods from 1 set of Monte Carlo replicates with estimated normal, lognormal, gamma, and Gumbel distributions.

For the normal, gamma, and lognormal distributions we used maximum likelihood parameter estimates, and we used the method of moments estimators of the Gumbel distribution parameters. For each set of 999 replicates, we get a different set of parameter estimates for each distribution. Figure 1 shows an example of a histogram from 1 set of 999 log-likelihoods with the estimated normal, lognormal, gamma, and Gumbel distributions. Even if the choice of distribution is the correct one, there is some error in the parameter estimates that plays a role in the ability of the approach to accurately estimate the p-value. While we used the moments estimator for the Gumbel distribution, which is very easy to compute, it is possible that the maximum likelihood estimates would have given slightly better results. The approach hinges on the ability of the estimated parameters to approximate the far tail of the true distribution of the statistic under the null, using only a relatively small number of randomly generated data sets. If the tail of the proposed distribution corresponds well to the far tail of the "gold standard" empirical distribution based on 100,000,000 random data sets, then the approach will be successful.

The idea now is to use the fitted distribution function to obtain a p-value. The p-value is calculated by finding the area under this distribution that is to the right of the observed test statistic. For this to work, it is important that the right tail of this function is similar to the right tail of the true distribution that is represented by the gold standard distribution from the 100,000,000 replicates. In order to check this, we used the cumulative distribution function (cdf) of each fitted distribution to find the critical value of the log-likelihood ratio corresponding to the nominal α-level. We then ranked each critical value among the 100,000,000 log-likelihood ratios in the gold standard distribution to find the true probability of rejecting the null at that critical value. We call this the rejection probability, and for the test to be unbiased, the expected value of this rejection probability must be equal to the nominal (desired) α-level. For each type of distribution, we did this 1000 times which resulted in 1000 critical values and, therefore, 1000 rejection probabilities. The average of these rejection probabilities is an estimate of the true (actual) α-level, which is then compared with the nominal α-level.

**Figure 2:**
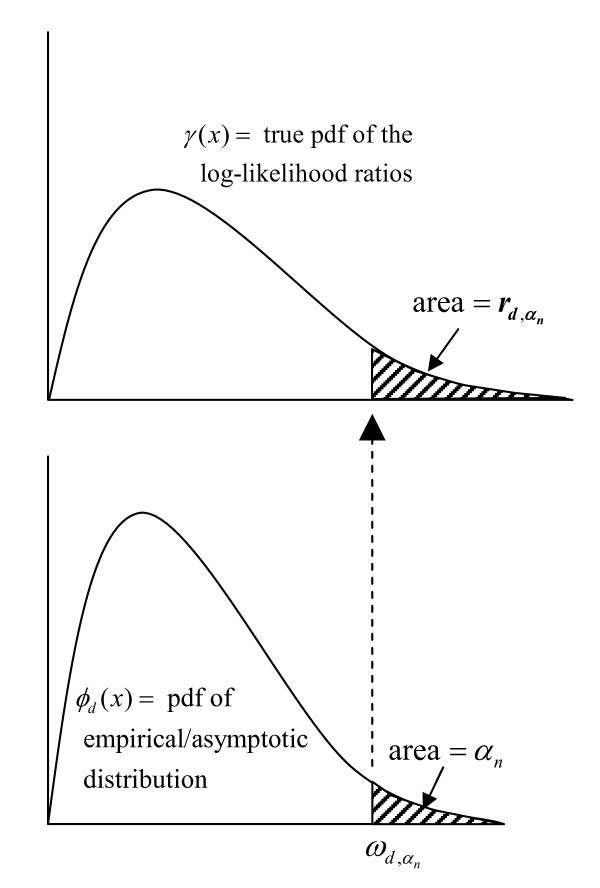
**Schematic for finding the rejection probability. **First find the critical value $\omega_{d,\alpha_{n}}$ from the fitted distribution, then use that value to find $r_{d,\alpha_{n}}$, the 'true' probability of rejecting the null hypothesis in the 'gold standard' pdf obtained from the 100,000,000 replicates under the null.

Here we present a formal description of this process; a schematic diagram is shown in Figure 2. Let $\phi_{d}(x)$ be the probability density function (pdf) of the distribution, *d*, obtained by using the log-likelihood ratios generated from the Monte Carlo replicates to estimate the parameters for *d*. Here we use *d *= normal, lognormal, gamma, and Gumbel. Let $\Phi_{d}(x)$ be the associated cdf. For the nominal α-level, $\alpha_{n}$, and each distribution, we first find the critical value $\omega_{d,\alpha_{n}}$ for which $1-\Phi_{d}\left(\omega_{d,\alpha_{n}}\right)=\int_{\omega_{d,\alpha_{n}}}^{\infty }\phi_{d}\left(x\right)dx=\alpha_{n}$; that is, we find $\omega_{d,\alpha_{n}}=\Phi_{d}^{-1}\left(1-\alpha_{n}\right)$. Note that $\omega_{d,\alpha_{n}}$ is the value for which the area under $\phi_{d}(x)$ and to the right of $\omega_{d,\alpha_{n}}$ is $\alpha_{n}$. Now, let γ(x) be the true pdf of the log-likelihood ratios and let Γ(x) be the corresponding cdf. Then $1-\Gamma \left(\omega_{d,\alpha_{n}}\right)=\int_{\omega_{d,\alpha_{n}}}^{\infty }\gamma \left(x\right)dx=r_{d,\alpha_{n}}$ is the rejection probability associated with $\alpha_{n}$ from distribution *d*. Of course, Γ(x) is unknown, and here we use the observed 100,000,000 replicates as a proxy. Effectively, $1-\Gamma \left(\omega_{d,\alpha_{n}}\right)=\frac{1}{100,000,000}\sum_{i=1}^{100,000,000}I\left(llr_{i}>\omega_{d,\alpha_{n}}\right)=r_{d,\alpha_{n}}$ where *llr*_*i *_is the *i*^th ^log-likelihood ratio in the gold-standard distribution. The average of the 1000 $r_{d,\alpha_{n}}$'s is our estimate of the true α-level, when the nominal α-level is $\alpha_{n}$. Note that the α-level found using Monte Carlo hypothesis testing, which we denote $\alpha_{MC,\alpha_{n}}$ is proven theoretically to be correct, so that $\alpha_{MC,\alpha_{n}}\equiv \alpha_{n}$.

In addition to the baseline set-up, we repeated the same experiment with different numbers of cases, different maps, different probability models, different maximum circle sizes, and different numbers of Monte Carlo replicates. We also repeated the experiment adding a temporal dimension. The combinations that we used are summarized in Table 1. United States 3-digit zip code populations were obtained from the 1990 United States Census [31]. For each set of conditions in Table 1, we performed the experiment using 99, 999, and 9999 Monte Carlo replicates to generate the fitted distribution. We chose 5 nominal α-levels ($\alpha_{n}$ = 0.05, 0.01, 0.001, 0.0001, 0.00001). All scan statistics were calculated using the SaTScan™ software.

**Table 1:** Combinations of settings used; bold indicates baseline settings.

	Number of cases	Region	Probability Model	Maximum cluster size
Spatial combinations	**600**	**NE counties**	**Poisson**	**50% population**
	600	NE counties	Bernoulli	50% population
	600	NE counties	Poisson	1 county
	600	US 3-digit zip codes	Poisson	50% population
	6	NE counties	Poisson	50% population
	6000	NE counties	Poisson	50% population
	60000	NE counties	Poisson	50% population

Space-time combinations	600	NE counties, 60 days	Poisson	50% population, 7 days
	600	NE counties, 60 days	Space-time permutation	50% population, 7 days

## Results

### α-levels

**Figure 3:**
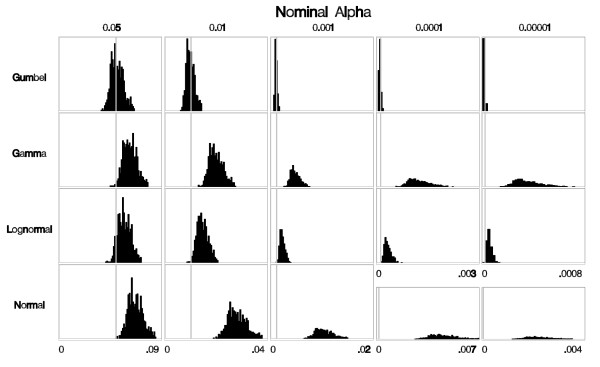
**Histograms of the rejection probabilities obtained from each of the fitted distributions. **The vertical gray lines indicate the nominal α-level. For α = 0.05, α = 0.01, and α = 0.001, the scales are the same within those columns with the scale marked at the bottom of each column. For α = 0.0001 and α = 0.00001, the scale is different for the normal distribution than for the other 3 distributions, as is indicated at the bottom of the histograms.

**Figure 4:**
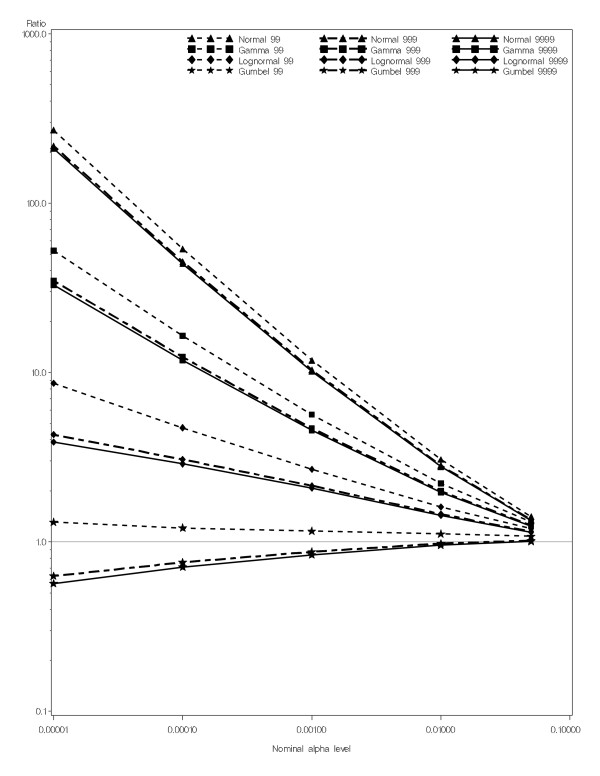
Ratio of estimated α-levels to nominal α-levels for 4 distributions and different numbers of Monte Carlo replicates used to estimate the parameters for each distribution.

The most important evaluation criterion in classical hypothesis testing is to ensure that the α-level (type I error) is correct. For the baseline experimental set-up, each histogram in Figure 3 shows 1000 rejection probabilities, one for each set of 999 Monte Carlo replicates generated under the null, for each fitted distribution. The mean of these rejection probabilities is an estimate of the true α-level achieved through this process. Note that in order to maintain the correct α-level, it is enough for the mean of the distribution to equal α, while the variance around α is irrelevant. Therefore, to maintain the correct α-level, it is sufficient that the rejection probabilities are centered around the desired α-level. Among the distributions assessed here, the rejection probabilities from the Gumbel approximation are centered around the nominal α-levels; for the other distributions the rejection probabilities are centered to the right of the nominal α-level. Thus, using any of these distributions other than the Gumbel distribution to approximate the underlying distribution of the spatial scan statistic results in anti-conservatively biased α-levels, or in other words, p-values that are too small. This can also be seen in Figure 4, where we show a plot of the ratio of the estimated true α-level to the nominal α-level for each distribution. Figure 4 shows that the Gumbel approximation has very little bias. When 999 or 9999 Monte Carlo replicates were used the slight bias is conservative, whereas the bias from all other distributions is large and anti-conservative.

The above results are all based on the baseline experimental setting. For the other settings, the true α-levels are presented in Tables 2 and 3 for the Gumbel distribution, showing the robustness of this method. Overall, the Gumbel approximation works quite well. The α-levels are slightly conservative whenever 999 or 9999 Monte Carlo replicates were used, and slightly anti-conservative with 99 replicates. The bias becomes increasingly conservative with an increasing number of replicates, and this conservatism is more pronounced in the space-time settings. The worst spatial results using the Gumbel distribution were for the very extreme scenarios when there are only 6 disease cases in all of the northeastern United States, when there is a non-negligible conservative bias. This is probably due to the extremely small sample size, and can be viewed as a worst case scenario. When the maximum scanning window size was limited to contain at most one county, there is a non-negligible anti-conservative bias. In this one-county experiment, every single county is evaluated as a potential cluster, but two or more counties are never combined to form a cluster. We are still taking the maxima when calculating the test statistic but it is a maxima over only 245 rather than tens of thousands of potential clusters. This may explain why the Gumbel extreme value distribution does not work as well in this scenario.

**Table 2:** Estimated α-levels for the Gumbel approximation for different parameters, corresponding to five nominal α-levels.

					Nominal alpha				
Number of cases	Maximum circle size	Region	Probability Model	Number of Monte Carlo replicates	0.00001	0.0001	0.001	0.01	0.05
6	50%	NE counties	Poisson	99	0.000003	0.00004	0.0006	0.008	0.051
				999	0.000001	0.00002	0.0004	0.007	0.048
				9999	0.000001	0.00002	0.0004	0.007	0.047

600	50%	NE counties	Poisson	99	0.000013	0.00012	0.0012	0.011	0.054
				999	0.000006	0.00008	0.0009	0.010	0.051
				9999	0.000006	0.00007	0.0008	0.010	0.050

600	50%	NE counties	Bernoulli	99	0.000014	0.00013	0.0012	0.011	0.053
				999	0.000007	0.00008	0.0009	0.010	0.050
				9999	0.000007	0.00008	0.0008	0.010	0.047

600	50%	US 3 digit zip codes	Poisson	99	0.000014	0.00013	0.0012	0.011	0.054
				999	0.000007	0.00008	0.0009	0.010	0.052
				9999	0.000006	0.00008	0.0009	0.010	0.051

600	1 county	NE counties	Poisson	99	0.000033	0.00022	0.0016	0.012	0.053
				999	0.000020	0.00016	0.0012	0.011	0.051
				9999	0.000018	0.00015	0.0018	0.011	0.050

6000	50%	NE counties	Poisson	99	0.000013	0.00012	0.0011	0.011	0.053
				999	0.000007	0.00008	0.0009	0.010	0.051
				9999	0.000006	0.00007	0.0008	0.010	0.050

60000	50%	NE counties	Poisson	99	0.000013	0.00012	0.0011	0.011	0.054
				999	0.000006	0.00007	0.0009	0.010	0.051
				9999	0.000006	0.00007	0.0008	0.009	0.050

**Table 3:** Estimated α-levels for the Gumbel approximation for different parameters for the space-time scan, corresponding to five nominal α-levels.

						Nominal alpha				
Number of cases	Maximum circle size	Maximum cluster length	Region	Probability Model	Number of Monte Carlo replicates	0.00001	0.0001	0.001	0.01	0.05
600	50%	7 days	NE counties	Space-time Permutation	99	0.000003	0.00004	0.0006	0.008	0.051
					999	0.000006	0.00007	0.0009	0.010	0.053
					9999	0.000003	0.00005	0.0007	0.009	0.051

600	50%	7 days	NE counties	Space-time Poisson	99	0.000002	0.00005	0.0007	0.010	0.051
					999	0.000006	0.00007	0.0008	0.010	0.051
					9999	0.000003	0.00004	0.0006	0.008	0.049

We also evaluated the other three distributions using all of the settings, and the bias was similar to, and as bad as, the results shown in Figures 3 and 4 (data not shown).

### Statistical power

**Figure 5:**
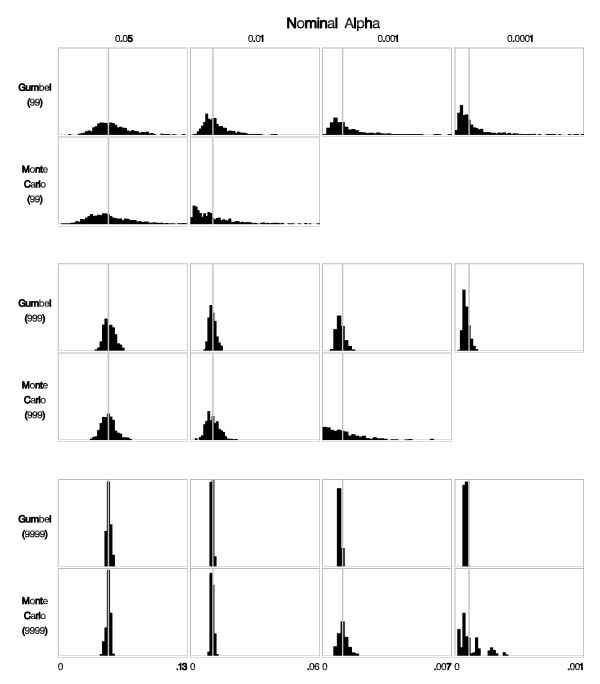
Histograms of the rejection probabilities obtained using the Gumbel approximation and from Monte Carlo hypothesis testing based on 99, 999, or 9,999 Monte Carlo replicates.

Statistical power is another important evaluation criterion. While the variance in the probabilities depicted in Figure 3 do not influence the α-level, a larger variance will slightly reduce the statistical power of the test, as implied by the proof of Jöckel [27]. We informally compared the power for the Gumbel approximation to the power obtained from Monte Carlo hypothesis testing by looking at the variance of the rejection probabilities used to calculate α-levels. Using the parameter set from the baseline experimental settings, Figure 5 shows the same type of histograms of the rejection probabilities as in Figure 3, but only for the Gumbel approximation and from Monte Carlo hypothesis testing, and using variable numbers of Monte Carlo replicates. The figure suggests that the variance in the rejection probabilities from the Gumbel approximation is smaller than the variance in the rejection probabilities from Monte Carlo hypothesis testing. Numerically, the ratio of the standard deviation of the rejection probabilities from the Gumbel approximation to the standard deviation of the rejection probabilities from Monte Carlo hypothesis testing is less than 1 when the same number of replicates are used for both methods, indicating that the scan statistic has greater power when the Gumbel approximation is used than with traditional Monte Carlo hypothesis testing. When we use 9999 replicates for Monte Carlo hypothesis testing and only 999 replicates for the Gumbel approximation this ratio is about 1; the same is true if 999 replicates are used for Monte Carlo hypothesis testing and 99 replicates are used for the Gumbel approximation. This suggests that in this example, 10 times as many replicates are required in order to get about the same power with Monte Carlo hypothesis testing as with the Gumbel approximation. Approximately the same relationship was observed in the space-time and other spatial settings.

## Discussion

We have shown that the Gumbel distribution can be used to obtain approximate p-values for the spatial and space-time scan statistics with great accuracy in the far tail of the distribution. This can be done using far less computation than required by the traditional method based on Monte Carlo hypothesis testing. As a rule of thumb, we suggest using at least 999 random Monte Carlo replicates to estimate the parameters of the Gumbel distribution, when possible, but the approach also works with a smaller number of replicates.

A key question is then when to use Monte Carlo hypothesis testing versus Gumbel based p-values. If the primary interest is in 0.05 and 0.01 alpha levels, or if the data set is small so that it is easy to generate and calculate the test statistic for hundreds of thousands of simulated replicas, then traditional Monte Carlo hypothesis testing works well, and the benefit of Gumbel based p-values is at most marginal. However, there are several instances in which the Gumbel approximations offer a clear advantage.

If the same number of replicates is used, then the Gumbel approximation has higher power than Monte Carlo hypothesis testing. When the number of replicates divided by the desired alpha level is large, the difference in power is marginal, but when it is small, there is a clear advantage of the Gumbel approximation. More specifically, the Gumbel approximation with one-tenth the number of replicates used by Monte Carlo hypothesis testing provides approximately the same statistical power, while using one-tenth of the computing time. Although there is some bias with the Gumbel approximation, the bias is small and, in most cases, conservative.

The most important benefit of the Gumbel approximation is its ability to calculate very small p-values with a modest number of simulated replicates. For example, as shown in Figure 4, p-values on the order of 0.00001 can be conservatively calculated with only 999 random replicates by using the Gumbel approximation, while it would require more than 99,999 replicates to get the same precision from Monte Carlo hypothesis testing.

The attempts to calculate p-values with the normal, lognormal and gamma distributions all resulted in anti-conservatively biased α-levels. The bias from these approximations was so large that we do not recommend their use to approximate p-values for spatial or space-time scan statistics.

The circular purely spatial scan statistic and the space-time scan statistic are only two examples of the many types of scan statistics. Other types include the elliptical shaped spatial scan statistics [32], non-parametric irregular shaped spatial scan statistics [33-35], as well as spatial and space-time scan statistics for ordinal [36] and exponential data [37,38]. While we have not tested the Gumbel approximation for other types of scan statistics, these statistics are all maxima and generating p-values for any of them relies on Monte Carlo hypothesis testing. It would be reasonable, then, to evaluate whether p-values for these other scan statistics could also be approximated with the Gumbel distribution.

The method used here of fitting a distribution to the statistics obtained from the Monte Carlo replicates can be applied to any other application in which Monte Carlo hypothesis testing is used and where very small p-values are required or where computing time is limited. There is no reason to expect the Gumbel distribution to work well in all situations, however. In this particular example it makes sense intuitively because the scan statistic generated in each replicate is a maximum over many circles and the Gumbel distribution is a distribution of maxima. Other applications may lend themselves naturally to a different choice of distribution.

To summarize, in applications in which the precision of small p-values is not important, we suggest using Monte Carlo hypothesis testing to obtain the p-values for the spatial scan statistic. In applications in which the precision of p-values is important or where each replicate takes a long time to complete, the Gumbel based p-values are often advantageous for reasons of both computational speed and statistical power. To facilitate its use, Gumbel based p-values have been added to version 9 of the freely available SaTScan software, which can be downloaded from http://www.satscan.org.

## Competing interests

The authors declare that they have no competing interests.

## Authors' contributions

AA programmed the simulations, analyzed the simulation results, and drafted the manuscript. KK and MK conceived of the idea, provided guidance, and helped draft the manuscript. All authors read and approved the final manuscript.
